# Barriers to accessing HIV pre‐exposure prophylaxis for Medicare‐ineligible people in Melbourne, Australia: analysis of patients attending the PrEPMe Clinic

**DOI:** 10.5694/mja2.51455

**Published:** 2022-03-12

**Authors:** Vincent J Cornelisse, Jude Armishaw, Mike Catton, Dean Murphy, Edwina J Wright

**Affiliations:** ^1^ Alfred Hospital Melbourne VIC; ^2^ Monash University Melbourne VIC; ^3^ Victorian Infectious Diseases Reference Laboratory Melbourne VIC; ^4^ Peter Doherty Institute for Infection and Immunity University of Melbourne Melbourne VIC; ^5^ Kirby Institute UNSW Sydney Sydney NSW; ^6^ Burnet Institute Melbourne VIC

**Keywords:** HIV, Sexually transmitted diseases, Prevention and control, Social justice, Homosexuality

## Competing interests

Vincent Cornelisse has received speaker’s fees and advisory board fees from Gilead Sciences, and advisory board fees from ViiV Healthcare. Edwina Wright reports receipt of grants from Gilead Sciences (free study drug for the VicPrEP study, compensation to her institution for chairing a nursing education session and for attending an advisory board meeting, and uncompensated attendance for attending two Gilead meetings regarding listing of Truvada on the Australian Pharmaceutical Benefits Scheme); grants from Gilead Science and Merck Sharp and Dohme outside the submitted work; and financial support from Gilead Sciences, Abbott Laboratories, Janssen‐Cilag, Boehringer Ingelheim, ViiV Healthcare, and Merck Sharp and Dohme.

to the editor: People without Medicare coverage cannot access Pharmaceutical Benefits Scheme (PBS)‐subsidised human immunodeficiency virus (HIV) pre‐exposure prophylaxis (PrEP) or associated clinical care. Rates of HIV infection diagnosis are disproportionately higher among overseas‐born gay and bisexual men compared with Australian‐born gay and bisexual men.^1^ In response, in June 2020, the Alfred Hospital and the Victorian Infectious Diseases Reference Laboratory established the free PrEPMe Clinic for Medicare‐ineligible people. Data were collected using proformas after patients provided verbal consent (Alfred Health Ethics Committee approval No. 656/18).

The first 100 consecutive patients were all born overseas (Box). Melbourne’s only public sexual health clinic referred 65 patients. Almost all patients were male, all patients had sex with men and reported a median of three sexual partners in 3 months at baseline; 76 patients inconsistently used condoms for anal sex. Fifty‐eight patients reported previous sexually transmissible infections (STIs); STIs were diagnosed in 12/100 patients at baseline, a rate similar to that found in Medicare‐eligible PrEP users.^2^ Thirty‐four patients had previously accessed HIV post‐exposure prophylaxis (PEP), and 49 patients had previously unsuccessfully attempted to obtain PrEP. The reported barriers to access mainly included costs of medical appointments and pathology, and difficulties navigating Australia’s health care system.

Box 1 Demographic characteristics, immunodeficiency virus (HIV) acquisition risk, and prior efforts to obtain pre‐exposure prophylaxis (PrEP) in the first 100 consecutive patients to attend the PrEPMe HIV prevention clinic at the Alfred Hospital in Melbourne, Australia*

**Table:** 

	Values
Total number of patients	100
**Demographic characteristics**	
Region of birth	
Asia	47
Latin America	31
Europe	14
Other	8
Age, years, median (IQR)	28 (26–31)
Gender	
Cisgender male	96
Transgender female	4
Visa status	
Student visa	62
Working visa	34
Other	4
**Referral sources**	
Melbourne Sexual Health Centre	65
Word of mouth	16
Other^†^	13
Unknown	6
**HIV risk at initial clinical assessment**	
Sexual partners (3 months), median (IQR)	3 (1–5)
Condom use for anal sex (3 months)	
Always	24
Mostly or sometimes	60
Never	13
Not applicable	1
Unknown	2
Previous STIs (ever)	
Yes	58
No	42
Previous STIs (ever, specific STIs)	
Gonorrhoea	35
Chlamydia	21
Syphilis	21
Other^‡^	5
STIs diagnosed at baseline	
Chlamydia only	6
Other^§^	6
**Previous attempts at HIV risk reduction**	
Previous use of PEP	
Yes	34
No	57
Unknown	9
Previous unsuccessful attempts to obtain PrEP	
Yes	49
No	46
Unknown	5
**PrEP commencement by 3‐month follow‐up**	
Commenced PrEP	87
Local pharmacy	65
Online	19
Online order did not arrive, then purchased at pharmacy	3
PrEP not commenced	6
Online order did not arrive	3
Other^¶^	3
Lost to follow‐up	7

COVID‐19 = coronavirus disease 2019; IQR = interquartile range; PEP = post‐exposure prophylaxis; STIs = sexually transmissible infections.

* Enrolment dates: 1 June 2020 to 26 October 2020.

† Includes general practices, internet search, “PrEP Access Now” Facebook page, Alfred Hospital PEP program.

‡ Includes herpes simplex virus, *Mycoplasma genitalium*, hepatitis B virus.

§ Includes syphilis, hepatitis B virus, both chlamydia and gonorrhoea.

¶ Includes lost prescription, no sex due to COVID‐19.

All patients received a non‐PBS PrEP prescription. At 3‐month follow‐up, 87 patients had commenced PrEP. Local pharmacies supplied PrEP at cost price (A$40–55 per month) or free to patients with financial hardship; other patients purchased PrEP online (US$20–30 per month) or obtained free PrEP online using assistance coupons (www.pan.org.au; Box). Most patients who ordered PrEP online experienced delivery delays of 4–6 weeks, leaving them at risk of HIV infection.

We report that Medicare‐ineligible gay and bisexual men and transgender women were at high risk of HIV infection, yet faced significant financial barriers to accessing PrEP. PrEP uptake has been associated with significant population‐level declines in incident HIV infection in Australia.^3^ Australia’s Eighth National HIV Strategy aims for virtual elimination of HIV transmissions by 2022,^4^ and to achieve this goal, Australia must provide universally subsidised PrEP medication and clinical services, irrespective of Medicare status.^5^ Medicare‐ineligible gay and bisexual men often already attend publicly funded sexual health clinics for free HIV/STI testing and treatment, as reported here. In a high income country like Australia, the additional cost of providing universally subsidised PrEP care would likely be lower than treating preventable new HIV infections, with an estimated lifetime cost of more than US$350 000 per HIV infection diagnosis.^6^
